# A practical individualized radiation precaution based on the dose rate at release time after inpatient ^131^I ablation therapy

**DOI:** 10.1371/journal.pone.0251627

**Published:** 2021-05-21

**Authors:** Sangwon Han, Soyoung Jin, Seon Hee Yoo, Hyo Sang Lee, Suk Hyun Lee, Min Ji Jeon, Jin-Sook Ryu

**Affiliations:** 1 Department of Nuclear Medicine, Asan Medical Center, University of Ulsan College of Medicine, Seoul, Korea; 2 Department of Nuclear Medicine, Nowon Eulji Medical Center, Eulji University School of Medicine, Seoul, Korea; 3 Department of Nuclear Medicine, Gangneung Asan Hospital, University of Ulsan College of Medicine, Gangneung, Korea; 4 Division of Nuclear Medicine, Department of Radiology, Kangnam Sacred Heart Hospital, Hallym University College of Medicine, Seoul, Korea; 5 Department of Endocrinology, Asan Medical Center, University of Ulsan College of Medicine, Seoul, Korea; IPATIMUP/i3S, PORTUGAL

## Abstract

**Introduction:**

Retained radioactivity of ^131^I after ablation therapy largely differs in each patient according to factors including the amount of remnant thyroid tissue, renal function, and use of recombinant human thyroid-stimulating hormone. To reduce unnecessary restriction of patient’s daily life after inpatient ^131^I ablation therapy, we propose a practical individualized method for radiation precaution based on dose rate at release time.

**Methods:**

We evaluated 215 patients with differentiated thyroid cancer who underwent inpatient ^131^I ablation therapy following total thyroidectomy. Effective dose equivalent rates at 1-m distance were measured upon release (EDR_R_) on day 2 and during delayed whole-body scan (EDR_D_) visits on day 6‒8 after ^131^I administration. The biexponential model was designed to estimate total effective dose equivalent to others. To assess conservativeness of our model, EDR_D_ estimated by our model was compared with measured EDR_D_. EDR_R_-based periods of precaution not to receiving 1 mSv of radiation exposure were estimated and compared with those based on administered radioactivities on American Thyroid Association (ATA) recommendations.

**Results:**

The EDR_R_ ranged from 1.0–48.9 μSv/hr. The measured EDR_D_ were equal to or lower than estimated EDR_D_ in all patients, except for one, indicating that our model is sufficiently conservative. According to our model, no subjects needed additional daytime restriction after release. The maximum permissible times for public transportation use were longer in all patients compared with those based on administered radioactivities. Nighttime restriction periods were significantly shorter than those based on administered radioactivity; median periods requiring sleeping apart were 0 (range, 0‒5), 4 (range, 1‒14), and 3 (range, 2‒13) days after release in patients treated with radioactivity doses of 2.96, 5.50, and 7.40 GBq, respectively, needing 8, 16, and 19 additional days, respectively, based on administered radioactivity.

**Conclusions:**

Radiation safety instructions using proposed method based on EDR_R_ of individual patient could safely reduce the burden of radiation precaution.

## Introduction

Radioactive iodine (RAI) therapy is an integral part of the standard care for differentiated thyroid cancer (DTC) [1]. For DTC patients with intermediate or high risk of recurrence following total thyroidectomy, RAI ablation therapy is recommended [2–4]. Moreover, when ^131^I used is more than 1.22 GBq (33 mCi) for RAI therapy, which is equivalent to a dose limit of >5mSv, several days of hospitalization in a licensed facility is mandatory [5, 6]. According to the US Nuclear Regulatory Commission (NRC) regulations, Title 10 of the Code of Federal Regulations part 35.75 [6], licensed facilities can only release patients treated with RAI therapy from their control if the radiation exposure to any other individual near the released patients is not likely to exceed 5 mSv, and if any individual is likely to receive >1 mSv of radiation, they should provide radiation safety instructions to patients to help maintain radiation exposure as low as reasonably achievable (ALARA).

The Society of Nuclear Medicine guideline [7] do not explicitly define precautionary isolation periods after release of patients treated with RAI therapy, but the NRC [6] and National Council on Radiation Protection and Measurements (NRCP) 155 [8] provide general operating equations for calculating radiation precaution periods. The American Thyroid Association (ATA) [9] and International Atomic Energy Agency (IAEA)/International Commission on Radiological Protection (ICRP) guidelines [10], which adopted the results from a study by Barrington et al. [11], provide restriction periods based on administered radioactivity. Additionally, ATA and NRC suggest individualized calculation of periods for radiation safety precaution taking into account various patient-specific factors including the administered ^131^I activity, effective half-lives, and thyroid uptake fractions [6, 9]. Regarding this, Liu et al. [12] reported that the actual restrictive periods for radiation precaution were significantly shorter than what was described in the ATA or ICRP recommendation based on administered radioactivity (i.e., 3 days of sleeping apart from pregnant women and children is actually required for a patient treated with 3.70 GBq [100 mCi] of RAI compared to the 13 days recommended by the ATA). Although fitting of biokinetic models with at least six measurement of whole-body dose rate conducted by this study could provide truly-individualized radiation safety guidance, it is unlikely to be performed in real-world nuclear medicine facilities due to increased risk of radiation exposure to medical staff and inconvenience. Therefore, the current practice regarding radiation precaution is largely differed across physicians partly due to the presence of either too simple (based on administered radioactivity) or too sophisticated (fitting biokinetic model) methods [13]. Also, the pattern of practice around the world with regard to the release of patients from hospital after RAI therapy is quite varied, so are dose limits and dose constraints adopted by governments around the world.

In the practice of RAI therapy in our country, the measurement of effective dose equivalent rate (EDR) at 1 m upon release is mandatory to decide whether patients can be released (if less than 70 μSv/hr) [9] and whether verbal and written instruction on radiation safety should be provided for any individual near the patient not to receive >1 mSv of radiation exposure (if above 20 μSv/hr) [6]. In this study, we formulated a simple and practical model based on the EDR upon release after inpatient RAI ablation treatment in patients with DTC to provide individualized post-release radiation safety precaution instructions to prevent exposure of >1 mSv to any person. Then, safety of our proposed model was evaluated by comparing measured EDR with its estimate by our model on 6–8 days after RAI therapy, the time of ^131^I post-treatment whole-body scan visit.

## Materials and methods

### Patient population

Between March and November 2015, 238 consecutive patients with histologically proven DTC underwent inpatient high-dose RAI therapy and the measurement of EDR at our institution. This study was approved by the local Institutional Ethics Committee (Asan Medical Center, Seoul, Korea), and the need for informed consent was waived due to its retrospective nature (IRB No; 2016–1082).

All subjects underwent total or near total thyroidectomy. Patients received RAI treatment following thyroid hormone withdrawal (four to five weeks for levothyroxine and two weeks for triiodothyronine) or injection of rhTSH to attain a thyroid-stimulating hormone (TSH) serum level of ≥30 mIU/L. All patients were instructed to restrict iodine intake for two weeks prior to RAI therapy. RAI dose was determined based on their pathologic stage, tumor size, extrathyroidal extension, histologic subtype, and presence of distant metastasis as described in S1 Table. Patients were hospitalized in a licensed and isolated treatment room for RAI therapy, and they were instructed to drink 2–3 liters of water a day during hospitalization after RAI administration. Two days after RAI administration, they were released from the isolation room once the external dose rates at 1 m from the patient were below 70 μSv/hr, which is the prescribed dose rate for authorized patient release, so as not to exceed 5 mSv of radiation exposure to other individual [6]. Post-therapeutic ^131^I whole-body images were acquired at two days and six to eight days, respectively. Blood sampling for measurement of serum TSH, creatinine, thyroglobulin (Tg), and anti-Tg antibody (TgAb) level was done on the same day of RAI therapy prior to its administration: glomerular filtration rate (GFR) has been estimated using the Chronic Kidney Disease Epidemiology Collaboration (CKD-EPI) equations [14]. The laboratory measurement of serum TSH, Tg, and TgAb levels have been previously described [15, 16].

The administered radioactivities of ^131^I in the study population were as follows: 124 patients received 2.96 GBq (80 mCi; 106 with rhTSH and 18 with thyroid hormone withdrawal), 3 patients received 3.70 GBq (100 mCi) with rhTSH, 95 patients received 5.55 GBq (150 mCi; 4 with rhTSH and 91 with thyroid hormone withdrawal), and 16 patients received 7.40 GBq (200 mCi) with thyroid hormone withdrawal. Among all patients, we excluded 23 patients from the analysis; those who were released on day 1 or 3 after RAI administration (n = 7), those who received 3.70 GBq (n = 3) or 5.55 GBq of ^131^I with rhTSH (n = 4), who had follow-up RAI treatment for distant metastasis after prior ablation therapy (n = 8), and those who had struma ovarii (n = 1). Finally, a total of 215 patients were included in the analysis to determine effective dose equivalent to other individuals near the patients. The patients were divided into four groups: 103 patients treated with 2.96 GBq of RAI after the use of rhTSH, 17 with 2.96 GBq, 88 with 5.55 GBq, and seven with 7.40 GBq after thyroid hormone withdrawal.

### Dose rate measurement

The EDR (μSv/hr) were measured with a survey meter equipped with Geiger-Mueller tube (Inspector, S.E international) at 1 m from the anterior mid trunk of the upright patient at a height of 1 m above the floor after urination. The measurements were performed two times on day 2, at the time of release upon hospital discharge (EDR_R_), and on days 6‒8 during whole-body scan visit (EDR_D_). The background activities were measured to calculate net patient EDRs. The survey meter was calibrated in an accredited laboratory with a calibration error of at most 6.5%.

### Estimation of effective dose equivalent to others from the patient

Assuming that clearance of ^131^I is biexponential [6, 9], whole-body dose rate (μSv/hr) at the time *t* (d) after administration, *A*(*t*) can be approximated using the following equation adapted from that of Zanzonico et al. [17]:

$$A(t)=A(0)\cdot \left(F_{1}\cdot e^{-\frac{0.693t}{T_{e1}}}+F_{2}\cdot e^{-\frac{0.693t}{T_{e2}}}\right),$$ (1)

where *F*_*1*_ and *F*_*2*_ are the ^131^I uptake fractions of the thyroidal and extrathyroidal components, respectively. *T*_*e1*_ and *T*_*e2*_ are the effective half-life of thyroidal and extrathyroidal components, respectively. According to ATA model [9], *F*_*1*_ and *F*_*2*_ were defined as 0.02 and 0.98, respectively. *T*_*e1*_ and *T*_*e2*_ were defined as 7.3 and 0.76 days, respectively.

The thyroidal and extrathyroidal fractions of EDR_R_ were calculated as follows:

$$\frac{0.02∙e^{-\frac{{0.693t}_{release}}{7.3∙24}}}{0.02∙e^{-\frac{{0.693t}_{release}}{7.3∙24}}+0.98∙e^{-\frac{{0.693t}_{release}}{0.76∙24}}},$$ (2)

as the thyroidal fraction, *F*_*1*, *release*_

$$\frac{0.98∙e^{-\frac{{0.693t}_{release}}{0.76∙24}}}{0.02∙e^{-\frac{{0.693t}_{release}}{7.3∙24}}+0.98∙e^{-\frac{{0.693t}_{release}}{0.76∙24}}},$$ (3)

as the extrathyroidal fraction, *F*_*2*, *release*_ where *t*_*release*_ (hr) is the time of release after ^131^I administration. Therefore, with the assumption of point-source [9], EDR (μSv/hr) at distance *r* (m) and time *t’* (d) after release, EDR (r, t’) may be written using EDR_R_ as the following equation:

$$EDR\left(r,t^{\prime }\right)=EDR_{R}\cdot \frac{1}{r^{2}}\cdot \left(F_{1,\mathrm{release}}\cdot e^{-\frac{0.693t^{\prime }}{T_{e1}}}+F_{2,\mathrm{release}}\cdot e^{-\frac{0.693t\prime }{T_{e2}}}\right),$$ (4)

As our patients underwent 2 days of isolation, the contribution of internalized radioactivity to total radiation was apparently negligible [18–20]. If we can set aside the contribution of internal contamination to total radiation burden, the total effective dose equivalent (TEDE, mSv) of individuals exposed by a patient from time *t’* to an infinite time can be calculated as follows [12]:

$$TEDE=\int_{t\prime }^{\infty }OF∙EDR(r,t^{\prime })dt=1.44\cdot OF\cdot EDR_{R}\cdot 10^{‐3}\cdot 24\cdot \frac{1}{r^{2}}\cdot (F_{1,\mathrm{release}}\cdot T_{e1}\cdot e^{-\frac{0.693t\prime }{T_{e1}}}+F_{2,\mathrm{release}}\cdot T_{e2}\cdot e^{-\frac{0.693t\prime }{T_{e2}}}),$$ (5)

where *OF* is an occupancy factor which accounts for the fraction of time spent in the vicinity of a treated patient. *24* and *10*^*−3*^ are conversion factors for unit of hour to day and μSv to mSv, respectively.

To assess the conservativeness of our estimates, we compared the EDR_D_ measures and estimates. The EDR_D_ estimates were calculated using Eq (4) with the distance (*r*) of 1 m and *t’* of the time interval between EDR_R_ and EDR_D_ in days.

### Estimation of restriction periods for radiation safety precaution

Restriction periods were calculated through an iterative process to find the smallest *t’* which is necessary to limit radiation exposure of any person near the patient to less than 1 mSv, using Eq (5). The following combinations of OF and index distance were used based on ATA model [9]: (1) upon arriving home, the patient spends 6 hrs a day (OF, 0.25) at 1 m from family members in daytime; (2) at night time, the patient spends 8 hrs asleep (OF, 0.33) at a distance of 0.3 m; (3) at work, co-worker spends 8 hrs a day (OF, 0.33) at 1 m from the patient.

For travel time restriction to public transport, we assumed the patient to be at a distance of 0.3 m from fellow passengers. Based on Eq (5), the maximum permissible time (hr), that is *OF* multiplied by 24 to reach TEDE of 1 mSv from each day (*t’* = 0, 1, 2, and 3 days) until tomorrow (*t’*+1), was calculated.

The restriction periods in our results based on EDR_D_ were compared with those based on administered radioactivity indicated by the ATA practice recommendation. Of note, we compared our results for nighttime restriction with the restriction period indicated as “Sleep in a separate bed from pregnant partners, infant, or child” in the ATA recommendation calculated based on 1 mSv [9]. With regard to a dose of 2.96 GBq of ^131^I, we calculated the administered radioactivity based restriction periods using the following equation: *EDR (t)* = *EDR (0)* ∙ (0.02 ∙ $e^{-\frac{0.693t}{7.3}}$ + 0.98 ∙ $e^{-\frac{0.693t}{0.76}}$), where EDR(0) was calculated using a dose rate of 1.7 μSv/mCi∙hr at 1 m distance [9].

## Results

### Patient characteristics

The patient baseline characteristics were outlined in Table 1. The median age was 47 years (range, 12‒78) with one pediatric patient included. The median serum creatinine level and calculated GFR at the time of administration were 0.76 mg/dL (range, 0.3‒1.52 mg/dL) and 96 mL/min (range, 50‒143 mL/min), respectively. Thirty patients (14%, 30/215) had a positive serum anti-Tg antibody, and the stimulated Tg levels ranged from 0.08 to 909.00 ng/mL with a median value of 0.58 ng/mL in the remaining 185 patients.

**Table 1:** Patient characteristics.

	rhTSH	Thyroid hormone withdrawal		
Administered radioactivities, GBq (mCi)	2.96 (80)	2.96 (80)	5.55 (150)	7.40 (200)
*n*	103	17	88	7
Male sex (n)	25 (24.3%)	5 (29.4%)	21 (23.9%)	3 (42.9%)
Age (y)	50 (20–73)	40 (12–66)	42 (19–74)	57 (35–78)
Serum creatinine (mg/dL)	0.61 (0.57–0.87)	0.94 (0.60–1.33)	0.82 (0.55–1.52)	0.98 (0.68–1.06)
GFR (mL/min)	116 (89–131)	80 (50–107)	86 (56–129)	94 (62–113)
Serum Tg (ng/mL)*	0.30 (0.08–6.10)	1.30 (0.08–9.40)	0.98 (0.08–243.00)	41.80 (1.40–909.00)

Data are expressed as median (range) or number (proportion).

*Subjects with positive anti-Tg antibody were excluded.

GFR = glomerular filtration rate; rhTSH = recombinant human thyroid-stimulating hormone; Tg = thyroglobulin

**Fig 1:**
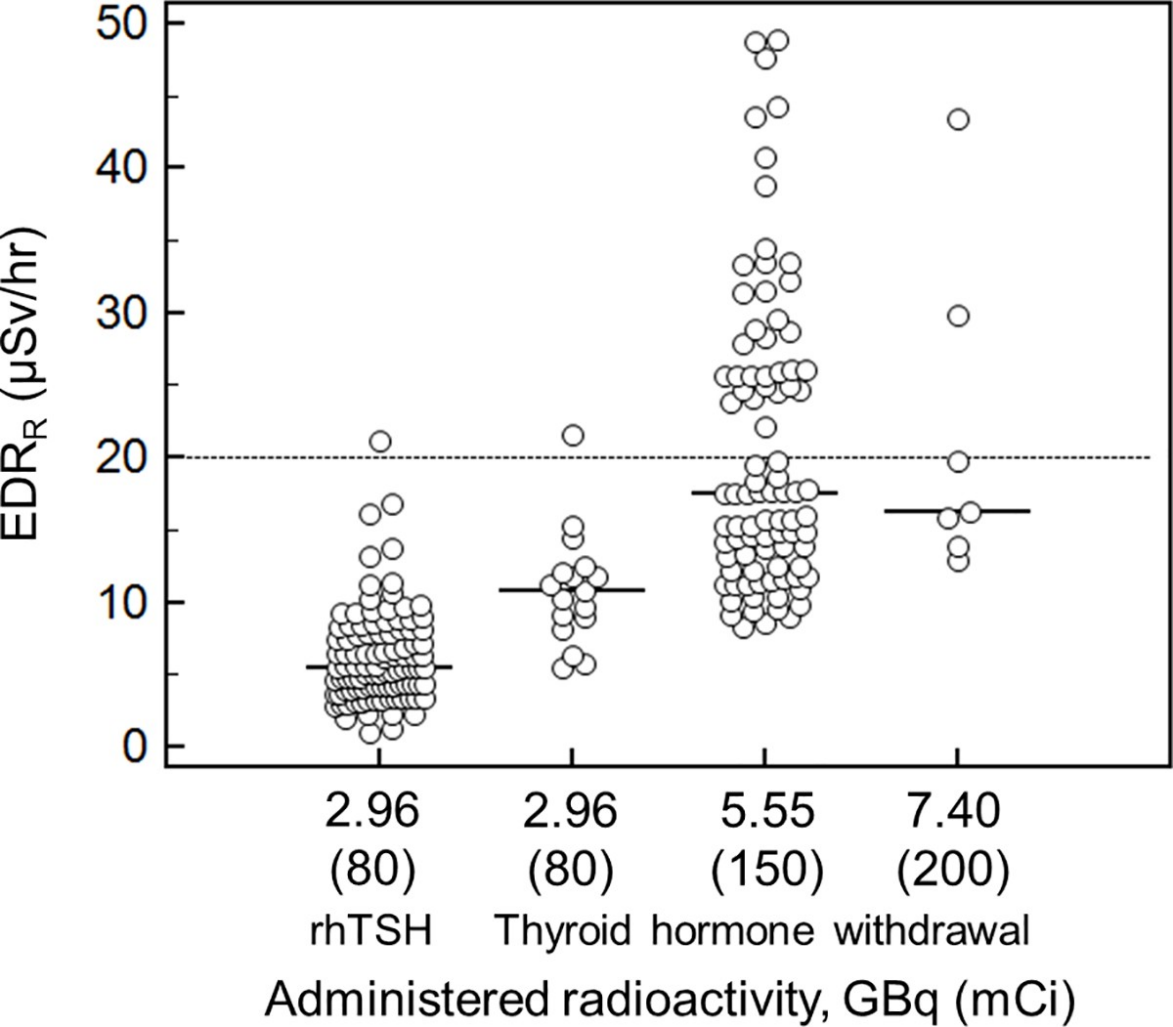
The effective dose equivalent rate at release (EDR_R_, day 2 after ^131^I administration) at 1 m according to the administered radioactivities. The dotted line represents a threshold providing the instruction on radiation safety.

The median EDR_R_ was 9.8 μSv/hr (range, 1.0‒48.9 μSv/hr) for all patients. The EDR_R_ stratified by administered radioactivities are presented in Fig 1. The hospitalization periods, *t*_*release*_, ranged from 39.5 to 42.3 hrs.

### Comparison between measures and estimates of EDR_D_

**Fig 2:**
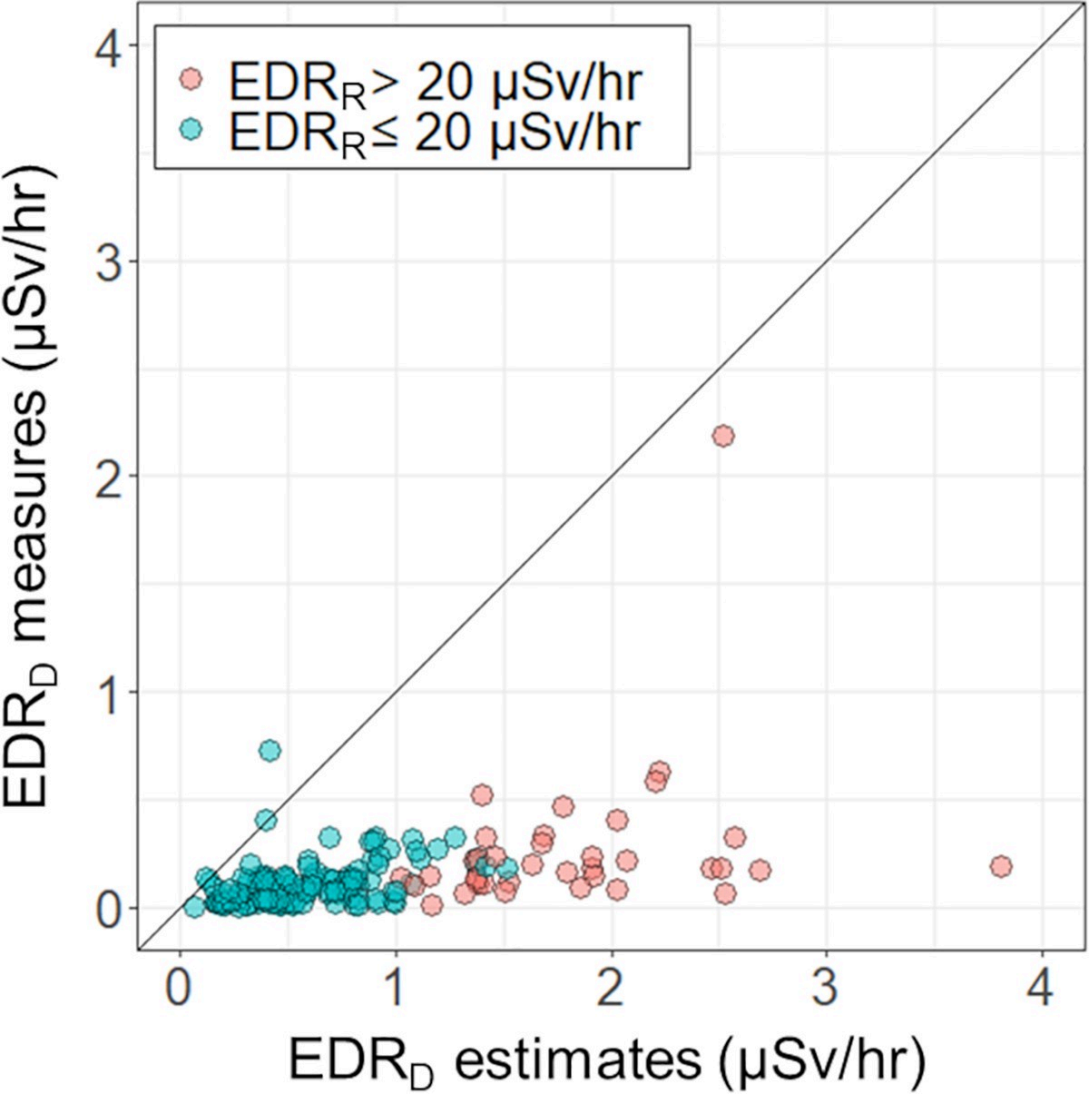
Comparisons between effective dose equivalent rates measured at a delayed scan time (EDR_D_ measures, days 6–8 after ^131^I administration) and estimates calculated using the EDR_R_ based on our model (EDR_D_ estimates). The line of equality is presented as a solid line. Red circles represent patients with EDR_R_ >20 μSv/hr who required verbal and written instructions on radiation precaution.

Fig 2 describes the comparison of EDR_D_ measures and estimates based on EDR_R_. The measured EDR_D_ ranged from 0.0 to 2.2 μSv/hr with a median value of 0.1 μSv/hr. The EDR_D_ measures were equal to or lower than the estimates of EDR_D_ (median, 0.6 μSv/hr; range, 0.1‒3.8) in all patients except one, whose EDR_D_ measure was 0.7 μSv/hr, whereas, the EDR_D_ estimate was 0.4 μSv/hr. In this patient, the administered radioactivity was 2.96 GBq with the use of rhTSH. Serum creatinine level and GFR were 0.57 mg/dL and 116 mL/min, respectively. On ^131^I whole-body image, a substantial amount of remnant thyroidal uptake of RAI was seen. The EDR_R_ was 4.8 μSv/hr for which a written safety instruction was not required.

### Restriction periods after release for radiation safety precaution

Based on EDR_R_, written radiation safety instruction was required (>20 μSv/hr) for 38 patients (18%): one patient (1%) with 2.96 GBq with rhTSH, one (6%) with 2.96 GBq with hormone withdrawal, 33 (38%) with 5.55 GBq, and 2 (29%) with 7.40 GBq.

None of the subjects treated from 2.96 to 7.40 GBq needed any restriction period after release for daytime with regard to (1) daytime contact with family member and (2) return to work. The maximum permissible time for public transportation use based on EDR_R_ were also less restrictive compared with those based on administered radioactivity as shown in Table 2.

**Table 2:** Maximum permissible time to use public transportation based on EDR_R_ compared to those based on administered radioactivity.

	Based on EDR_R_			Based on administered radioactivities		
Administered radioactivities, GBq (mCi)	2.96 (80)	5.55 (150)	7.40 (200)	2.96 (80)	5.55 (150)	7.40 (200)
Day 0 at release (h)	21.1 (6.2–24.0)	7.6 (2.7–16.2)	8.1 (3.1–10.3)	5.5	2.5	1.9
Day 1 (h)	24.0 (13.6–24.0)	16.7 (6.0–24.0)	17.8 (6.7–22.7)	11.8	5.0	3.8
Day 2 (h)	24.0	24.0 (11.6–24.0)	24.0 (13.0–24.0)	22.3	10.0	7.5

Data based on EDR_R_ are expressed as medians (ranges).

EDR_R_ = effective dose equivalent rates upon release (on day 2 after ^131^I administration; at a 1-m distance)

**Fig 3:**
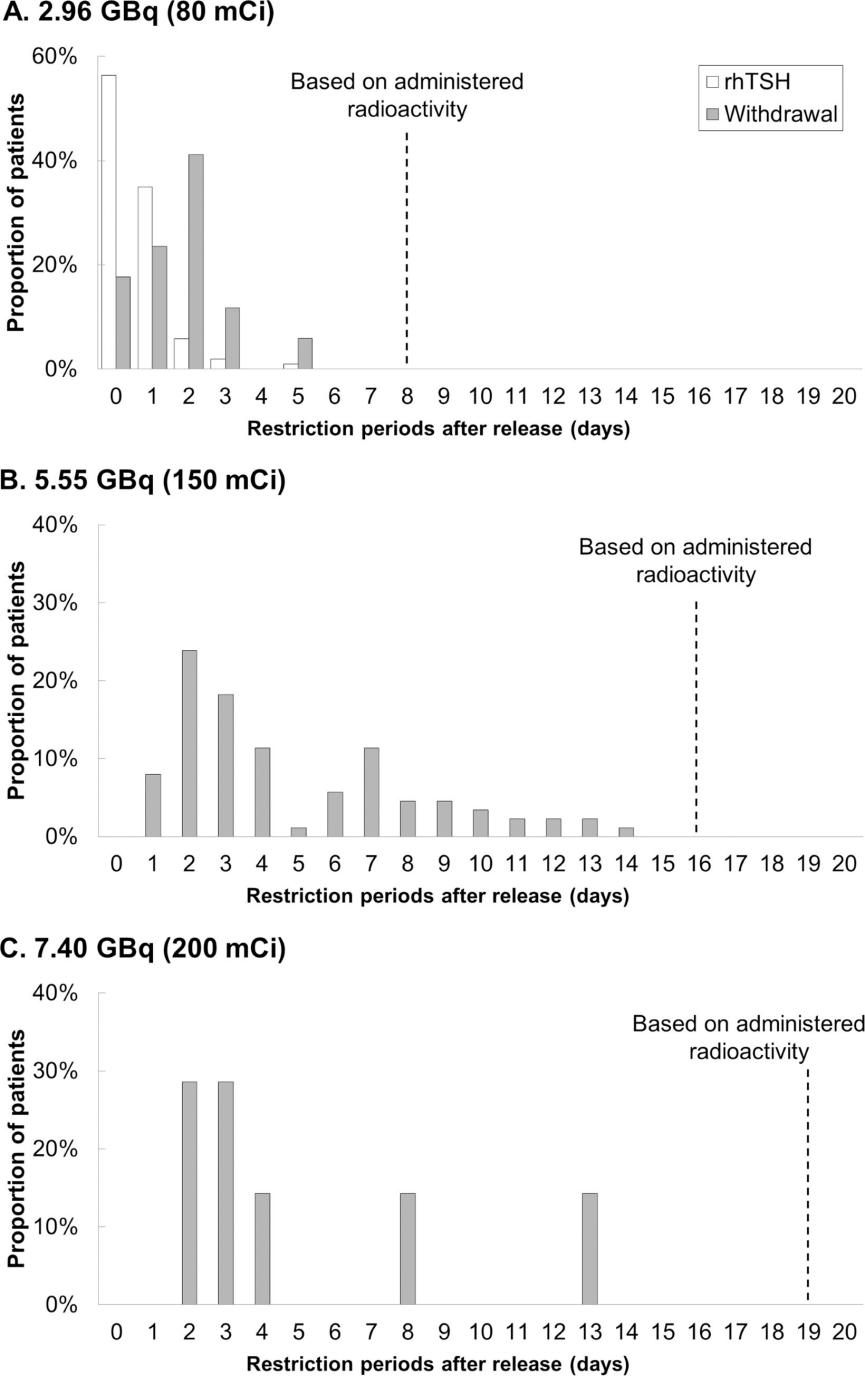
The histograms show the radiation restriction periods in nighttime with children or pregnant partner in patients stratified by their administered radioactivities of 2.96 (A), 5.55 (B), and 7.40 GBq (C) of RAI. The dashed vertical lines represent the restriction periods based on administered radioactivity.

For nighttime restriction on sleeping with family members in a separate bed, up to 14 days of restriction periods were needed for all patients, which were below those based on administered radioactivity (Fig 3). Particularly in the 2.96 GBq group, median days requiring restriction were 0 days (range, 0‒5 days) and 2 days (range, 0‒5 days) for those prepared with rhTSH and hormone withdrawal, respectively, compared to the 8 days based on administered radioactivity. Of note, 97.1% (100/103) of patients prepared with rhTSH and 82.4% (14/17) of patients prepared with hormone withdrawal needed only ≤2 days of restriction. A median of 4 days (range, 1‒14 days) and 3 days (range, 2‒13 days) of nighttime restrictions were required for patients treated with radioactivity doses of 5.55 and 7.40 GBq, respectively, which were much less than the administered radioactivity based precaution periods of 16 and 19 additional days after release, respectively. Less than or equal to four days of sleeping apart from their family members were required in 61.4% (54/88) and 71.4% (5/7) of patients treated with 5.50 and 7.40 GBq, respectively.

## Discussions

The major findings of the present study are as follows: first, the EDR_R_ largely differed even in patients with an equivalent administered radioactivity of RAI advocating the necessity of individualized instruction; second, the radiation precaution requirements based on the EDR_R_ were far below those based on administered radioactivity stated in the current recommendation based on administered radioactivity; third, our results were, nevertheless, sufficiently conservative as illustrated by comparing the EDR_D_ measures to the EDR_D_ estimates based on our model.

For the evaluation of radiation precaution requirements based on EDR_R_, two sets of thyroidal uptake fractions and effective half-lives are available either from the NRC [6] or ATA model [9]. The initial thyroidal uptake fraction of ^131^I is 0.05 for the NRC model, which is based on an upper limit of post-thyroidectomy state, where 0.02 is described in the ATA model. In a previous study by Liu et al. [12], the mean uptake of remnant thyroid tissue is 1.8% of the administered radioactivities. Both models defined the effective half-life of the thyroidal fraction as 7.3 days, which is based on the biologic half-life of an adult thyroid tissue of 80 days [6]. With regard to the effective half-life for the extrathyroidal component, the NRC model used the iodine clearance rates in normal subjects of 0.32 days [6], while the ATA model set 0.76 days on the basis of those in hypothyroid status. Both models assumed the patient as a point source of ^131^I. The NRC model uses the specific gamma-ray constant of 2.2 R∙cm^2^/mCi∙hr, which is a value for unshielded point source, whereas the ATA model adopts a shielded point-source model using an effective gamma-ray constant of 1.7 R∙cm^2^/mCi∙hr for which distribution and photon attenuation in the patient’s body are taken into account [5]. Because the NRC model is generally considered as overly conservative [20], we adopted the ATA model to formulate the periods of radiation safety precautions. We found that our results were comparable to the suggested guidelines in the previous work by Culver and Dworkin [21], which were based on the observed radiation exposure rate of up to 7 days following hospital discharge and also conservative when compared to EDR_D_.

**Fig 4:**
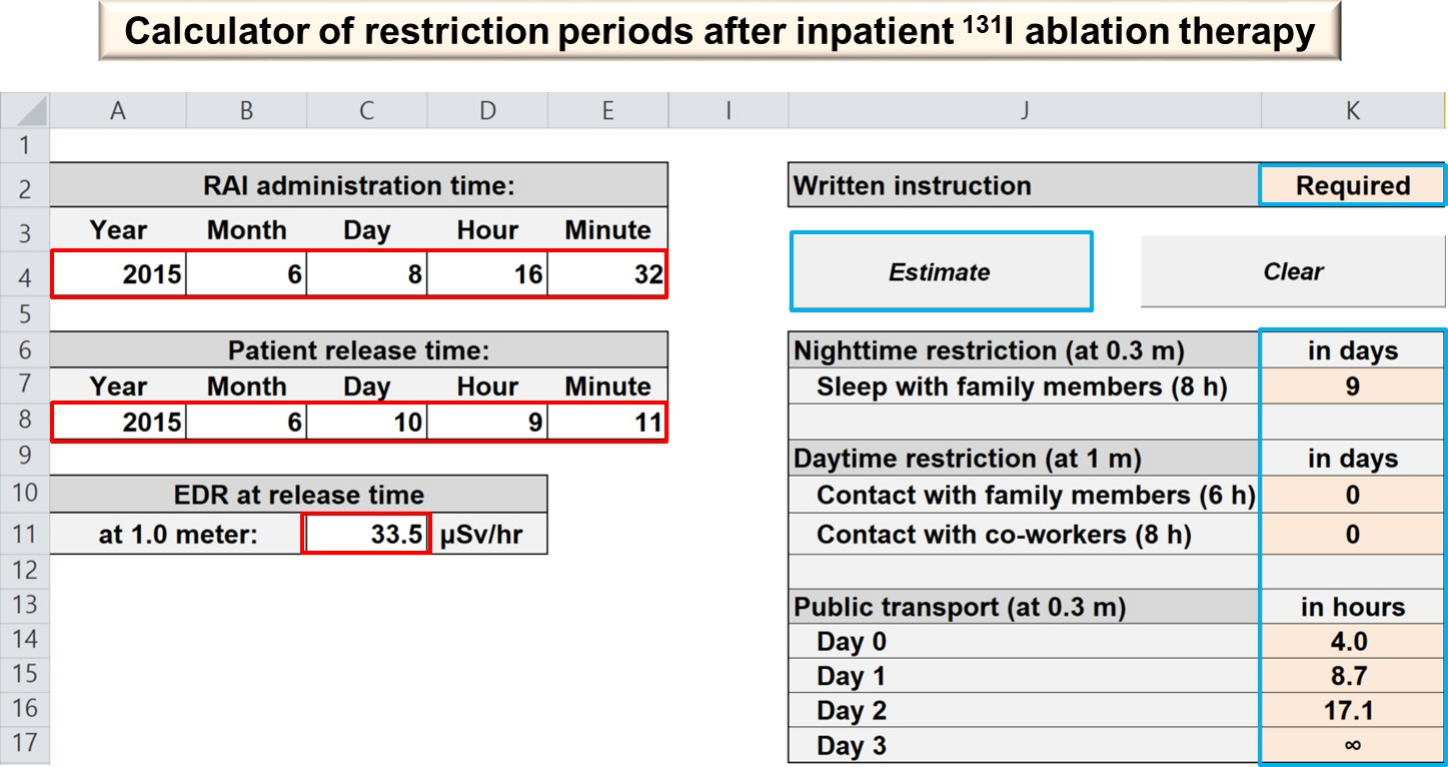
An example of spreadsheet for calculation of restriction periods after inpatient ^131^I ablation therapy (S1 File): Data input screen (marked in red) and generated restriction periods after “Estimate” button (marked in blue).

There are several possible explanations to our restriction periods being shorter than those based on administered radioactivity indicated by the current ATA practice recommendation [9]. First, the actual effective half-life of remnant thyroid tissue is shorter than 7.3 days. It has been noted in previous articles that ^131^I clears more rapidly from remnant thyroid tissue [11, 12, 22–24]. The shorter half-life of RAI can be explained by radiation-induced thyroiditis which can cause the release of radioiodinated Tg and thyroxine [25]. Second, using the effective half-life of 0.76 days for extrathyroidal component might be overestimated. Liu et al. [12] reported that the effective half-life for extrathyroidal component were 0.53 days. Similarly, Barrington et al. [11] found the corresponding value of 0.50 days. Third, the point-source assumption of the ATA model overestimates the actual dose rates at an index distance of 1 m [26]. Rather than a point source, the ^131^I distribution in the whole body is better modeled as a line source [27]. The actual values of EDR_R_ at 1 m in our study are lower than those expected in the ATA model. Accordingly, our estimates of EDR at 0.3 m using “inverse-square law” based on EDR_R_ (at 1 m) are also lower compared to those from the ATA model. Finally, thyroidal uptake fraction in our study population might be lower than 0.02 of the ATA model. Our institution is a tertiary medical center with high surgical volume, and thyroidectomy is performed by a surgical specialty group. The amount of residual thyroid tissue is related to surgical volume and specialization [28, 29]. Moreover, Yap et al. [29] discovered that the median RAI uptake in remnant thyroid tissue was less than 2% in patients with thyroidectomy performed by specialized surgeons. Of note, approximately half of our study population used rhTSH, which prevents delayed excretion of RAI in extrathyroidal components from hypothyroid state [30], while it may cause higher uptake of RAI in remnant thyroid tissue. Although the potential effect of rhTSH is not considered in effective half-life and thyroidal fraction, our model based on EDR_R_ is more predictive of necessary required radiation precautions compared with the model based on administered radioactivities. As compared with the one-size-fits-all and over-restrictive instructions, our model for inpatient therapy could provide patient-specific guidance that is able to enhance patients’ compliance and, at the same time, decrease inconvenience or reduce the social cost in a negligible risk increase for radiation exposure. For easy application to clinical practice, the spreadsheet for estimation of restriction periods of each patient was provided in Fig 4 and S1 File. By simply inputting the time data for RAI administration and time and EDR upon patient release on a simple spreadsheet we provided in S1 Dataset, every nuclear medicine facilities on which RAI treatment is performed can easily apply and calculate customized individual radiation safety instructions.

There are several limitations to our study. First, radiation exposure from internal contamination was not considered. Although the NRC guidelines provide a rough estimate of the maximum dose from internal exposure, we did not consider the contribution of internal exposure in our TEDE model owing to negligible internal contamination [18–20] and 2 days of isolation of our study population. Nevertheless, potential ingestion of ^131^I contaminants can be vary between patients and is a concern for small children [5]. Second, in this retrospective study, we could not generate a patient-specific excretion curve because we did not measure the EDR at multiple time points. However, measuring a series of EDR during or after hospitalization increases the risk of radiation exposure to medical staff and, thus, is not practical. Third, our results are not applicable to patients who underwent follow-up treatment for residual or recurrent disease following ablation therapy. For these patients, thyroidal uptake fraction of ^131^I is zero, and the dose rates decrease monoexponentially [11]. Likewise, caution is necessary when applying our results to patients with substantial thyroid remnant tissue (i.e., subtotal thyroidectomy) or high metastatic burden which demonstrates iodine avidity. Fourth, our model may not be suitable to patients with renal dysfunction. Although the half-life of 0.76 days for the extrathyroidal component in the ATA model is attributed to decreased renal function due to hypothyroid state, increased retention of RAI in these patients requires a prolonged period of radiation precaution. Further studies on EDR_R_-based radiation precautions are warranted for patients who undergo follow-up treatment and those with substantial thyroidal uptake fraction or renal dysfunction. Incorporating a post-treatment whole-body scan to the model would provide clues.

## Conclusions

The EDR_R_ after ^131^I ablation therapy varied in a wide range of patients with DTC. Therefore, radiation precaution instruction after inpatient ^131^I therapy should be patient-specific. Our model based on EDR_R_ provided a simple and practical way for patient-specific instruction of radiation safety precaution which was less restrictive compared with those based on administered radioactivity but was sufficiently conservative. We believe that this model can be easily applied in the practice of radiation safety guidance in RAI facilities, and enhance compliance as well as reduce the burden of patients and social cost.

## Supporting information

S1 Table Determination of therapeutic dose. (DOCX)

S1 File Spreadsheet for calculation. (XLSM)

S1 Dataset Individual patient data. (XLSX)
