# Supplementary material for: A practical individualized radiation precaution based on the dose rate at release time after inpatient 131I ablation therapy
Source: PLoS One. 2021 May 21;16(5):e0251627. doi: 10.1371/journal.pone.0251627 (PMC8139448; doi:10.1371/journal.pone.0251627)
Supplement: S1 Table — (DOCX) [file pone.0251627.s001.docx]

**S1 Table**. Determination of therapeutic dose (GBq)

| Resection  margin | T | N0 | N0 | pN1a | pN1b | M1 |
| --- | --- | --- | --- | --- | --- | --- |
| Negative | pT1a | None | 1.11^a^ | 2.96 | 5.55 | 7.40 |
|  | pT1b | 1.11^a^ |  |  |  |  |
|  | pT2 |  |  |  |  |  |
|  | pT3 (-2.0 cm) |  |  |  |  |  |
|  | pT3 (2.1-4.0cm) | 2.96 | 2.96 |  |  |  |
|  | pT3 (4.1cm-) | 5.55 | 5.55 | 5.55 |  |  |
|  | pT4a |  |  |  |  |  |
|  | pT4b | 7.40 | 7.40 | 7.40 | 7.40 |  |
| Positive | pT1a | 1.11^a^ | 1.11^a^ | 2.96 | 5.55 | 7.40 |
|  | pT1b | 2.96 | 2.96 |  |  |  |
|  | pT2 |  |  |  |  |  |
|  | pT3 (-2.0cm) |  |  |  |  |  |
|  | pT3 (2.1-4.0cm) | 5.55 | 5.55 | 5.55 |  |  |
|  | pT3 (4.1cm-) |  |  |  |  |  |
|  | pT4a |  |  |  |  |  |
|  | pT4b | 7.40 | 7.40 | 7.40 | 7.40 |  |

Poor histologic subtype (Hurthle cell carcinoma, insular carcinoma, poorly differentiated carcinoma) was treated with 5.55 GBq

^a^Outpatient treatment
